# Linc00668 Promotes Invasion and Stem Cell-Like Properties of Breast Cancer Cells by Interaction With SND1

**DOI:** 10.3389/fonc.2020.00088

**Published:** 2020-02-14

**Authors:** Wenchang Qian, Yong Zhu, Mingming Wu, Qianying Guo, Zhengsheng Wu, Peter E. Lobie, Tao Zhu

**Affiliations:** ^1^Department of Oncology of the First Affiliated Hospital, Division of Life Science and Medicine, The CAS Key Laboratory of Innate Immunity and Chronic Disease, University of Science and Technology of China, Hefei, China; ^2^Department of Pathology, Anhui Medical University, Hefei, China; ^3^Tsinghua Shenzhen International Graduate School, Tsinghua-Berkley Shenzhen Institute, Tsinghua University, Shenzhen, China; ^4^Shenzhen Bay Laboratory, Shenzhen, China; ^5^Hefei National Laboratory for Physical Sciences at Microscale, University of Science and Technology of China, Hefei, China

**Keywords:** long non-coding RNA, metastasis, chemotherapy resistance, SND1, breast cancer

## Abstract

Long non-coding RNAs (lncRNAs) are reported to be involved in breast cancer progression. Herein, we observed that the expression of Linc00668 was increased in breast cancer compared to normal tissue. The patients with high Linc00668 expression exhibited an association with a higher metastatic risk. We demonstrated that forced expression of Linc00668 enhanced, whereas depletion of Linc00668 diminished invasion and self-renewal of breast cancer cells as well as resistance to doxorubicin (Dox). Further mechanistic studies revealed that Linc00668 associated with staphylococcal nuclease domain-containing 1 (SND1) and regulated the expression of downstream genes. Linc00668 depletion led to reduced expression of the downstream target of SND1 and further attenuated the self-renewal capacity of breast cancer cells. Our observations suggest that Linc00668 promotes metastasis, and chemotherapeutic resistance in breast cancer by interacting with SND1. Therefore, Linc00668 may serve as a potential therapeutic modulator in breast cancer treatment.

## Introduction

Breast cancer remains the most common cancer in women and among the leading causes of female cancer mortality globally (1). Despite many years of efforts to improve diagnosis and treatment, the mortality of breast cancer patients still remains unacceptably high; and which is predominantly caused by local recurrence or distant metastasis. The tumor bulk is understood to be heterogeneous with a small fraction of cancer cells, usually termed cancer stem cells (CSCs), cancer metastasis-initiating cells (CMICs), or tumor-initiating cells (TICs) (2). According to the literature, breast cancer stem cells (BCSCs) exhibit a higher capacity for epithelial-mesenchymal transition (EMT), self-renewal, and chemotherapeutic resistance (3, 4). BCSC play a pivotal role in the progression and recurrence of breast cancer, poising a major obstacle for effective breast cancer treatment (4). Thus, effective therapy targeting BCSCs needs to be exploited to provide an improved outcome for breast cancer patients.

Long non-coding RNAs (lncRNAs), RNA transcripts longer than 200 nucleotides, without protein-coding capacity, have been implicated in cancer progression (5, 6). Typically LncRNAs interact with RNAs, RNA binding proteins (RBPs), or chromatin DNA to regulate mRNA stability, protein activity/stability, as well as chromatin accessibility in multiple cellular processes (7–9) including cancer (10–15).

Linc00668 has been reported to promote cancer development in laryngeal squamous carcinoma (LSCC), oral squamous carcinoma (OSCC), and gastric cancer (GC) in previous studies (16–18). Recent studies suggest Linc00668 as a marker in breast cancer risk prediction and facilitating tumor growth (19, 20). However, the multiple role of Linc00668 in breast cancer remains to be further studied. In this study, we observed that higher levels of Linc00668 were associated with lymphatic metastasis in breast cancer patients. Forced expression of Linc00668 promoted cell invasion, stem-cell like property, and doxorubicin resistance in breast cancer cells. Mechanistically, Linc00668 bound to SND1 and facilitated the expression of downstream genes. Given this, Linc00668 may serve as a candidate target for BCSCs-based therapy in breast cancer.

## Materials and Methods

### Breast Cancer Cell Lines and Specimens

HMEC-hTERT, MCF-10A, MCF-7, T47D, MDA-MB-231, HS578t, and 293T cell lines were provided by the American Type Culture Collection (ATCC), and cultured as recommended. SUM149, SUM159 cell lines were kind gifts from Suling Liu's lab. MCF-7 DOX-R cell lines were maintained continuously in 10 μM of Doxorubicin. The specimens used in this study, included 54 breast cancer tissue samples and 20 benign mammary tissue samples, from patients who underwent surgery at the First Affiliated Hospital of Anhui Medical University, between 2009 and 2010. The clinical research protocol has been approved by the Biomedical Ethics Committee of Anhui Medical University.

### Plasmid Constructs and Lentivirus Production

The sequence of Linc00668 (NCBI accession number NR_034100.1) was cloned into pSin plasmid, kindly provided by Huafeng Zhang laboratory (University of Science and Technology of China). The shRNAs of Linc00668 and SND1 were cloned into pLKO.1 vector. The lentivirus production by Calcium phosphate in 293T cells was performed according to Trono's lab protocol (http://tronolab.epfl.ch). The infected cells with lentivirus plasmids were selected with 1~2 μg/ml puromycin for 48 h.

### RNA Isolation and qRT-PCR

Total RNA was extracted from cells or clinical samples using the Trizol reagent (Invitrogen) and converted to cDNA using RevertAid First Strand cDNA Synthesis Kit (Thermo Scientific Bio) according to the manufacturer's instructions. The cDNA samples were analyzed by quantitative real-time PCR using SYBR Green dye (TAKARA) on Stratagene Mx3000P (Agilent Technologies).

### Protein Extraction and Western Blot Analysis

Total protein was extracted from the cells using the modified RIPA lysis buffer. The Western blot analysis was performed as previously described (21) and imagined with the ImageQuant LAS4000 (GE Healthy). The primary antibodies from Santa Cruz used were as follows: anti-Nanog, 1:1000, sc-293121 (22); anti-Oct4, 1:1000, sc-5279 (22); anti-Sox2, 1:1000, sc-365823 (23); anti-SND1, 1:1000, sc-271590 (24, 25); anti-β-Actin, 1:5000, sc-8432 (26).

### Cell Function Assays and Flow Cytometry Analysis

Five thousand cells were plated into Polyhema (Sigma)-coated 6-well plates to induce sphere formation. The mammosphere culture medium was performed as previously described (27). A transwell assay was performed in uncoated 8 μm pore chambers (Corning Costar) for migration, and Matigel (Coring Costar) coated chambers for invasion, following the detailed protocol (28). Briefly, the cell suspension (5 × 10^5^/ml) was placed into the upper chamber in 0.2 ml of serum-free medium, with 10% FBS culture medium in the lower chamber, and incubated in 37°C. For MTT assay, 2,000 cells were seeded into 96-well plates per well in a final volume of 200 μl complete medium with different concentrations of Dox, and the OD570 was measured using the MTT reagent (Promega), after 5 days. The ALDE-FOUR assay was performed accordingly to the user guide of the ALDE-FOUR Kit (Stem Cell Technologies). The aldehyde dehydrogenase (ALDH) positive staining was detected by BD FACS Verse Flow Cytometer.

### Biotin Pull-Down and Immunoprecipitation Assay

For biotin pull-down assay, the sense or antisense biotin-labeled DNA oligomers targeting Linc00668 were incubated with the cell lysates and streptavidin-coupled agarose beads (Invitrogen) were used to enrich the Linc00668 complex 1 h after incubation. For the RNA immunoprecipitation assay, anti-IgG (Santa Cruz, sc-2025, 1:100), anti-SND1 (Santa Cruz, sc-271590, 1:100), and the Protein A/G agarose (Santa Cruz, sc-2003) were used following the detailed protocols from the Santa Cruz web site at www.scbt.com. All steps were performed in an RNase-free environment following the detailed descriptions (29).

### Statistical Analysis

All experiments were repeated at least three times. GraphPad Prism (San Diego, CA) was used for statistical analysis. Data were expressed as mean ± SD and analyzed using Student's *t*-test or two-way ANOVA test. *P* < 0.05 was considered as statistically significant.

## Results

### Elevated Expression of Linc00668 Is Associated With Higher Metastatic Capacity in Breast Cancer

**Figure 1:**
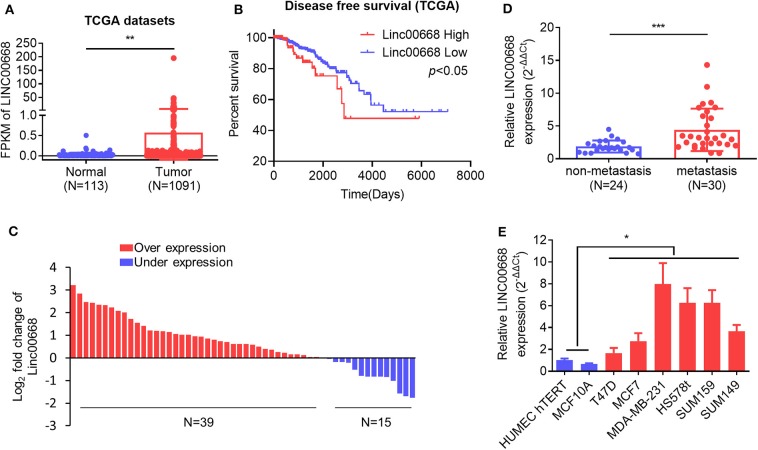
Linc00668 expression is increased in breast cancer. **(A)** The expression level (Fragments per kilobase of exon per million reads mapped, FPKM) of Linc00668 in benign breast tissue samples (*N* = 113) and breast cancer samples (*N* = 1,091) in TCGA database. **(B)** Kaplan-Meier analysis of the relationship of Linc00668 expression levels and disease free survival (DFS) of breast cancer patients. **(C)** Fold change of Linc00668 level in breast cancer tissues (*N* = 54) compared with the average of benign breast tissues (*N* = 19). **(D)** qRT-PCR analysis of the expression level of Linc00668 in non-metastasis cancer samples (*N* = 24) and metastatic cancer samples (*N* = 30). **(E)** qRT-PCR analysis of Linc00668 expression in 2 non-transformed cell lines and 6 breast cancer cell lines. qRT-PCR data were normalized to β-ACTIN and presented as 2^−ΔΔCt^ values relative to one of the normal tissues or one of the non-tumorigenic cell lines. Data are presented as the mean ± SD of three individual experiments, and error bars represent the SD. *p < 0.05; **p < 0.01; ***p < 0.001 (Student's *t*-test).

To assess the role of Linc00668 in the development of breast cancer, we first analyzed expression of Linc00668 in 113 normal breast tissue samples and 1,091 breast cancer samples from the TCGA datasets. A higher Linc00668 expression level was observed in the breast cancer tissue samples compared to the benign breast tissue samples (Figure 1A). Kaplan-Meier survival analysis showed that breast cancer patients with high expression of Linc00668 exhibited a shorter disease free survival compared with patients whose tumors expressed lower levels of Linc00668 (Figure 1B). Consistently qRT-PCR analyses of collected breast specimens of normal (*N* = 19) and tumorous (*N* = 54) tissue samples again revealed that Linc00668 expression was higher in 72.2% (39 of 54) of the cancer tissues compared to average of the normal tissues (Figure 1C). We further observed that higher levels of Linc00668 were associated with lymphatic metastasis in breast cancer patients (Figure 1D). The relationship between Linc00668 and other clinicopathological characteristics are summarized in Supplementary Table 1. We also examined the expression of Linc00668 in an array of mammary epithelial cell lines, including 2 non-transformed cell lines (HMEC hTERT, MCF-10A) and 6 breast cancer cell lines (T47D, MCF-7, SUM149, MDA-MB-231, HS578t, SUM159) by qRT-PCR (Figure 1E). The expression of Linc00668 was significantly higher in the cancer cell lines compared to the non-transformed cell lines. Hence, it may be suggested that Linc00668 is functionally involved in breast cancer development and progression.

### Linc00668 Promotes Breast Cancer Cell Invasion and Self-Renewal

**Figure 2:**
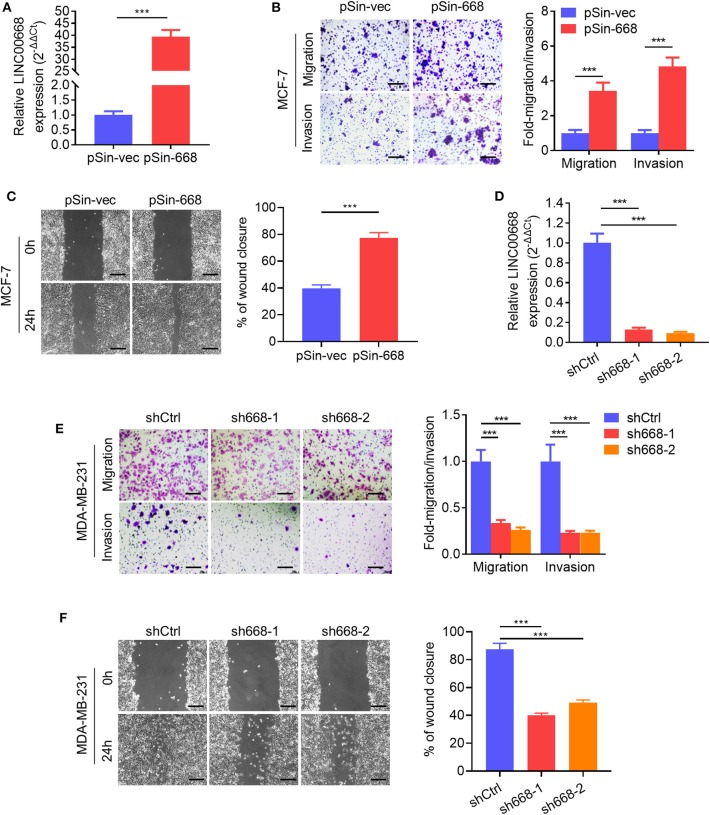
Linc00668 promotes the migrative and invasive capacity of breast cancer cells. **(A)** The expression levels of Linc00668 in MCF-7 cells stably transfected with pSin plasmid harboring Linc00668 sequence or pSin empty vector. **(B)** Transwell migration and invasion assays of MCF-7 cells with forced expression of Linc00668. Quantification of the migrated or invaded cell numbers per field was shown as fold changes relative to the control cells. **(C)** Wound healing assays of MCF-7 cells with forced expression of Linc00668. The percent wound closure was plotted and normalized to the width of wounds at 0 h. **(D)** The expression level of Linc00668 in MDA-MB-231 cells stably transfected with pLKO.1 plasmids harboring shRNAs of Linc00668 or pLKO.1 empty vector. **(E)** Transwell migration and invasion assays of the MDA-MB-231 derived cell lines. Quantification of the migrated and invaded cell numbers per field is shown as fold changes relative to control cells. **(F)** Wound healing assays of the MDA-MB-231 derived cell lines. The percent wound closure was plotted and normalized to the width of wounds at 0 h. Scale bars represented for 200 μm. Results are shown as mean ± SD. ***p < 0.001 (Student's *t*-test).

As higher Linc00668 levels have been suggested to be involved in the regulation of breast cancer metastasis, we further determined if Linc00668 could regulated breast cancer cell migration and invasion. Thus, the minimally invasive breast cancer cell line, MCF-7, was stably transfected with either the empty lentivirus plasmid (pSin-vec) or the plasmid carrying the Linc00668 sequence (pSin-668). The expression levels of Linc00668 were subsequently quantified by qRT-PCR (Figure 2A). As expected, forced expression of Linc00668 increased the migrative, invasive (Figure 2B), and wound closing (Figure 2C) capacity of MCF-7 cells compared to control transfected cells. In addition, either the empty vector pLKO.1 or the vector harboring Linc00668 shRNAs (sh668-1, sh668-2) was transfected into the highly invasive breast cancer cell line, MDA-MB-231; the silencing efficiency of shRNAs confirmed by qRT-PCR (Figure 2D). In contrast, Linc00668 depletion decreased the migrative, invasive, (Figure 2E) and wound closing (Figure 2F) of MDA-MB-231 cells.

**Figure 3:**
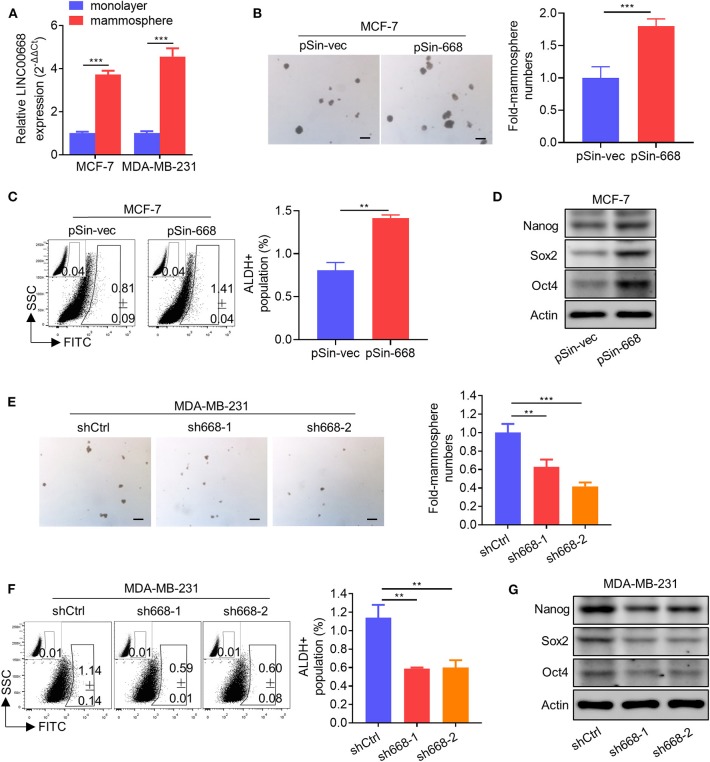
Linc00668 enhances the stem cell-like capacity of breast cancer cells. **(A)** Expression levels of Linc00668 were determined by qRT-PCR in MCF-7 and MDA-MB-231 cells by monolayer culture or suspension culture. **(B)** Representative images of mammosphere culture of respective MCF-7 derived cell lines. Quantification is shown as fold changes relative to control cells. **(C)** ALDH positive population was determined by ALDE-FLUOR assay and flow cytometric analysis in MCF-7 derived cell lines. **(D)** Western blot analysis of protein levels of stem cell markers in MCF-7 derived cell lines. β-Actin was used as internal control. **(E–G)** Suspension culture **(E)**, ALDE-FLUOR assay **(F)**, and Western blot analysis **(G)**, in respective MDA-MB-231 derived cell lines. Scale bars represented for 200 μm. Results are shown as mean ± SD. **p < 0.01; ***p < 0.001 (Student's *t*-test).

As invasive cancer cell phenotypes have been associated with characteristics of BCSCs (2, 4), we further investigated whether Linc00668 could modulate the stem cell-like properties of breast cancer cells. Using non-attached suspension culture (30), we measured the expression level of Linc00668 in MCF-7 and MDA-MB-231 cells either in suspension culture or monolayer culture by qRT-PCR and observed significantly higher expression of Linc00668 in mammospheres grown in suspension culture compared to the monolayer-cultured cells (Figure 3A). Consistently, forced expression of Linc00668 increased the size and number of mammospheres derived from MCF-7 pSin-668 cell compared to control cells (Figure 3B). Numerous studies have identified ALDH1 enzymatic activity as a marker for BCSCs (31–33). We therefore performed the ALDEFLUOR assay to determine whether Linc00668 modulated the ALDH1 positive cell population. Forced expression of Linc00668 in MCF-7 cells resulted in a significantly increased percentage of ALDH1 positive cells (Figure 3C). Consistently, we observed that the expression levels of stem cell markers, Nanog, Sox2, and Oct4, were markedly upregulated in cells with forced expression of Linc00668 compared to control cells (Figure 3D). Conversely, Linc00668 depletion in MDA-MB-231 cells decreased mammosphere formation (Figure 3E), the percentage of ALDH1 positive cells (Figure 3F), and Nanog, Sox2, Oct4, protein levels (Figure 3G). Collectively, these results demonstrated that Linc00668 enhanced stem cell-like characteristics in breast cancer cells.

### Linc00668 Promotes Resistance to Doxorubicin in Breast Cancer Cells

**Figure 4:**
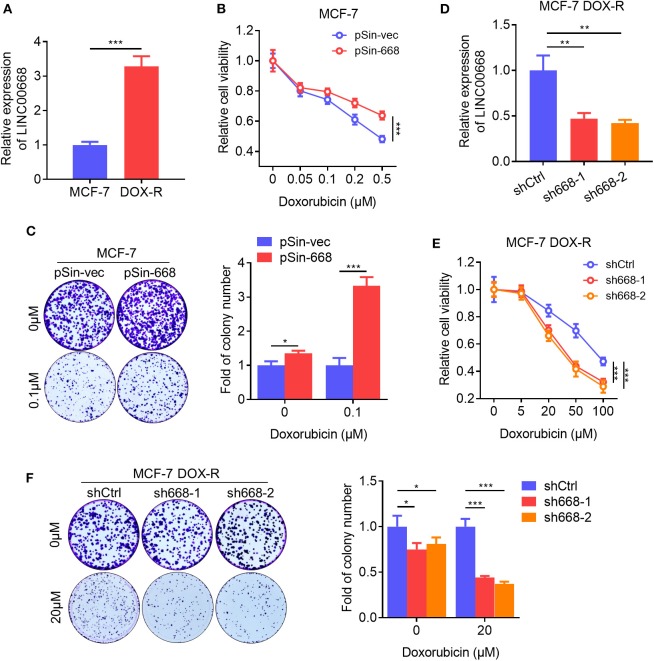
Linc00668 promotes doxorubicin resistance in breast cancer cells. **(A)** Expression level of Linc00668 of MCF-7 and MCF-7 DOX-R cells were determined by qRT-PCR. **(B)** Cell viabilities of MCF-7 pSin-668 or pSin-vec cells incubated with indicated doses of Dox analyzed by MTT assay. **(C)** Colony formation assay of MCF-7 derived cells in presence of Dox or vehicle. Quantification of the colony numbers presented as fold changes relative to the control cells. **(D)** qRT-PCR analysis of Linc00668 expression in MCF-7 DOX-R cells transfected shRNAs (sh668-1, sh668-2) or empty vector. **(E,F)** Cell viabilities **(E)**, colony formation assay with quantification of the colony numbers of MCF-7 DOX-R transfected shRNAs or control **(F)**. Results are shown as mean ±SD. *P < 0.05; **P < 0.01; ***P < 0.001 (Student's *t*-test, One-way ANOVA test).

BCSCs exhibit a higher capacity of resistance to multiple chemotherapeutic agents compared to bulk tumor cells (34). We observed that the expression level of Linc00668 was elevated in doxorubicin (Dox) resistant MCF-7 DOX-R cells compared to parental cells (Figure 4A). Hence, we further determined if Linc00668 regulated the sensitivity to doxorubicin in breast cancer cells. The cell viability and colony-formation capacity of MCF-7 vec and pSin-668 cells were assessed in presence of different concentrations of Dox. Forced expression of Linc00668 in MCF-7 cells resulted in increased cell viability (Figure 4B) and colony forming capacity compared to control cells in the presence of increasing concentrations of doxorubicin (Figure 4C). Conversely, MCF-7 DOX-R cells were transfected with a vector harboring Linc00668 shRNAs (sh668-1, sh668-2) or empty vector. Linc00668 expression levels in these two cell lines were analyzed by qRT-PCR (Figure 4D). We further showed that Linc00668 depletion significantly re-sensitized MCF-7 DOX-R cells to increasing amount of doxorubicin, as observed in both cell viability (Figure 4E) and colony formation assays (Figure 4F).

### Linc00668 Associated With SND1 to Stimulate the Expression of Its Downstream Targets

**Figure 5:**
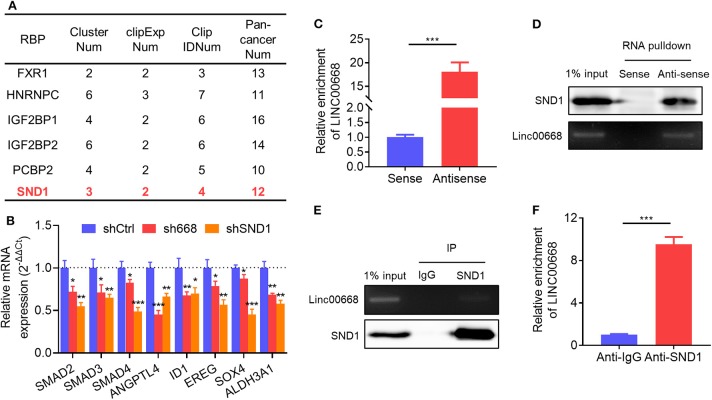
Physical association of Linc00668 and SND1. **(A)** RBPs predicted by starBase v2.0. **(B)** Expression of reported downstream genes of SND1 determined by qRT-PCR in MDA-MB-231 cells stably transfected shRNAs or pLKO.1 empty vector. **(C,D)** Linc00668 were immunoprecipitated using biotin-labeled anti-Linc00668 probes in MDA-MB-231 cells, and Linc00668 in pull-down RNA fraction were detected by qRT-PCR **(C)** and semi-qRT-PCR **(D)**. SND1 in above immunoprecipitates were detected by western blotting **(D)**. **(E,F)** SND1 were immunoprecipitated using anti-SND1 antibodies in MDA-MB-231 cells, and Linc00668 in immunoprecipitates were detected by semi-qRT-PCR **(E)** and qRT-PCR **(F)**. SND1 in above immunoprecipitates were detected by western blotting **(E)**. Results are shown as mean ± SD. *P < 0.05; **P < 0.01; ***P < 0.001 (Student's *t*-test).

Reports indicate that lncRNAs are involved in multiple aspects of cancer progression by interacting with RNA-binding proteins (RBPs) (29). Thus, we screened the RBPs which were predicted to physically interact with Linc00668 from the large-scale CLIP-Seq data by starBase v3.0 (http://starbase.sysu.edu.cn/). Several candidate RBPs were listed in Figure 5A. We then focused on the transcriptional activator, SND1, which was reported to promote breast cancer metastasis, and tumor initiation (35–38). qRT-PCR analysis showed that 8 of the reported downstream genes of SND1 were down-regulated in MDA-MB-231 cells with inhibition of Linc00668 (Figure 5B). To further verify the interaction of Linc00668 and SND1, a RNA immunoprecipitation (RIP) assay was carried out in MDA-MB-231 cells. The efficacy of anti-sense DNA probes was verified by qRT-PCR, and enriched the RNA chains of Linc00668 (Figure 5C) and also enriched the SND1 protein (Figure 5D). Sense DNA probes were used as controls. Reciprocally, the anti-SND1 antibody also enriched SND1 protein (Figure 5E) and Linc00668 (Figure 5F) in co-immunoprecipitation assays. As SND1 has been reported to directly bind to the promoters of SMAD2/3/4 for their transcriptional activation (38), we further determined if Linc00668 impacted the transcription of SMAD2/3/4 via SND1. Forced expression of Linc00668 in MCF-7 cells dramatically increased the expression of SMAD2, SMAD3, and SMAD4 (Supplementary Figure 1A), whereas Linc00668 depletion in MDA-MB-231 cells diminished the expression levels of SMAD2, SMAD3, and SMAD4 (Supplementary Figure 1B). Linc00668 shRNA (sh668) had limited effect on SMAD2/3/4 expression in MDA-MB-231 cells with SND1 inhibition (shSND1) (Supplementary Figure 1C), suggesting Linc00668 stimulated the transcription of SMAD2/3/4 via SND1.

### Functional Characterization of SND1 in Breast Cancer Cells

**Figure 6:**
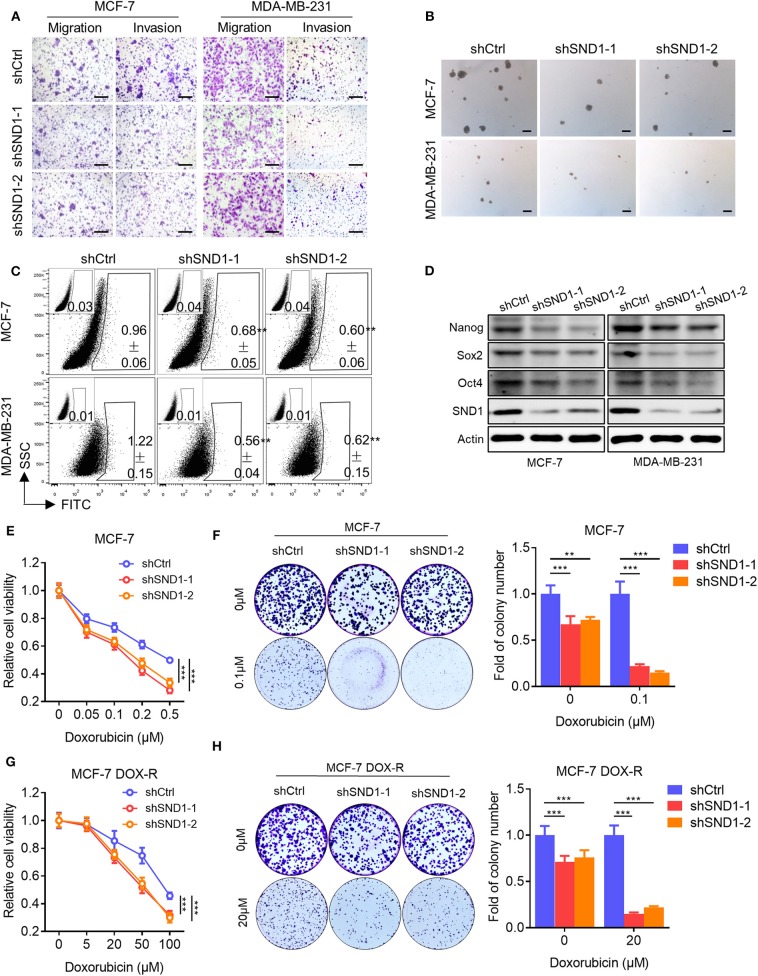
SND1 depletion represses breast cancer cell migration, invasion, stem cell-like capacity and doxorubicin resistance. **(A)** Transwell migration and invasion assays of MCF-7 (left) and MDA-MB-231 (right) cells with SND1 depletion. Scale bars represented for 200 μm. **(B)** Representative images of mammosphere culture of respective MCF-7 and MDA-MB-231 derived cell lines. Scale bars represented for 200 μm. **(C)** ALDH positive population of perspective stable cell lines determined by flow cytometric analysis. **(D)** Western blot analysis of protein expression of stem cell markers in MCF-7 (left) and MDA-MB-231 (right) derived cell lines. β-Actin was used as internal control. **(E)** Cell viability assay of MCF-7 cells transected with either vector or shRNA. **(F)** Colony formation assay of MCF-7 cells transected with either vector or shRNA in presence of Dox or vehicle. Quantification of the colony numbers is presented as fold changes relative to the control cells. **(G,H)** Cell viability assay **(G)**, colony formation assay **(H)** of MCF-7 DOX-R cells transected with either vector or shRNA. Results are shown as mean ± SD. **P < 0.01; ***P < 0.001 (Student's *t*-test, One-way ANOVA test).

As it was suggested above that SND1 mediated Linc00668 function in breast cancer cells, we also examined the function of SND1 in breast cancer cells. Lentivirus plasmids carrying SND1 shRNAs (shSND1-1, shSND1-2) or control plasmid were introduced into MCF-7 or MDA-MB-231 cells (Figure 6D). We found that SND1 depletion resulted in reduced migration and invasion of MCF-7 and MDA-MB-231 cells (Figure 6A, Supplementary Figure 2A). In addition, silencing of SND1 significantly reduced the number and size of mammospheres (Figure 6B, Supplementary Figure 2B) and the percentage of ALDH+ cells (Figure 6C, Supplementary Figure 2C) in MCF-7 and MDA-MB-231 cells. Consistently, SND1 depletion also resulted in reduced expression of Nanog, Sox2, and Oct4 (Figure 6D). SND1 depletion in MCF-7 cells also reduced cell viability and colony formation (Figure 6E) and increased the cellular sensitivity to doxorubicin treatment (Figure 6F). Further, we showed that SND1 depletion re-sensitized MCF-7 DOX-R cells to Dox (Figures 6G,H). Hence, reduced SND1 expression in breast cancer cells resulted in decreased invasive and self-renewal capacity and also increased sensitivity to Dox.

### SND1 Mediates the Function of Linc00668 in Breast Cancer Cells

**Figure 7:**
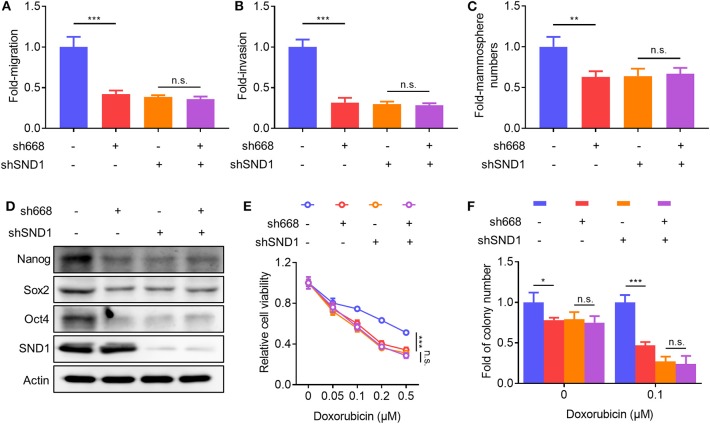
SND1 mediates the cellular effects of Linc00668. **(A–F)** MCF-7 shCtrl or shSND1 stable cell lines were infected with sh668 or pLKO.1 plasmid. Cell migration **(A)**, cell invasion **(B)**, mammosphere formation **(C)**, protein expression of stem cell markers and SND1 **(D)**, cell viability **(E)**, colony formation **(F)** were determined, respectively. Results are shown as mean ± SD. *P < 0.05; **P < 0.01; ***P < 0.001; n.s., not significant (Student's *t*-test, One-way ANOVA test).

To determine if Linc00668 promoted oncogenicity was mediated via SND1, lentivirus plasmids carrying Linc00668 shRNA (sh668-1) or control plasmid were infected to either MCF-7 cells with inhibition of SND1 or control transfected cells. As expected, Linc00668 inhibition had limited effect on migration (Figure 7A), invasion (Figure 7B), and mammosphere formation (Figure 7C) in cells with SND1 depletion. Linc00668 inhibition had limited effect on the protein levels of Nanog, Sox2, and Oct4 in cells with SND1 depletion (Figure 7D). We further observed that Linc00668 inhibition barely re-sensitized cells with SND1 depletion to increasing concentrations of doxorubicin as observed by viability (Figure 7E) and colony formation assays (Figure 7F). Hence, inhibition of Linc00668 in breast cancer cells suppressed SND1 mediated migration, invasion, self-renewal, and doxorubicin resistance.

## Discussion

Herein, we reported the elevated expression of Linc00668 in breast cancer tissues compared to benign breast tissues and which was associated with a higher metastatic capacity. We further observed that forced expression of Linc00668 increased cell invasion, self-renewal, and the percentage ALDH1 positive cell population. As cytotoxic treatment has often been reported to enrich BCSCs (39), as expected, Linc00668 expression was elevated in Dox resistant breast cancer cells and forced expression of Linc00668 promoted the development of the resistance to Dox. Thus, Linc00668 plays an important role in breast cancer progression including the development of chemotherapeutic resistance.

We further observed that Linc00668 associated with SND1 in breast cancer cells. SND1 has been reported to play an important role in epithelial-mesenchymal transition, tumor initiation, and tumor progression of breast cancer (36–38, 40), as well as in other types of cancer (41–44). SND1 is a promiscuous protein that interacts with proteins, RNA and DNA. The versatility of SND1 was reported as an interacting protein with transcription factors and co-regulators to participate the transcription of multiple genes in different cellular processes (38, 45). Other co-factors may participate in the transcript initiation complex with SDN1, such as STAT6, STAT5, and c-Myb (45). Besides the role of a transcription co-activator, the Tudor-SN domain of SND1 participates in pre-mRNA splicing (46), and the staphylococcal nuclease-like domains also act as bridging factors or RNA binding proteins to enhance the mRNA stability (47–49). Our findings suggest that Linc00668 plays an important role in cell invasion, self-renewal capacity, and Dox resistance by interacting with SND1 to facilitate the expression of several downstream genes. However, the exact mechanism utilized by Linc00668-SND1 interaction and its associated co-factors to elicit the transcriptional activity was not determined, which is warranted in the future study.

It was also reported that SND1 recognized the conserved Motif domains of SMAD2/3/4 promoters, inducing their transcription (38). Besides, pSMAD2/3 also in turn activates the transcription of SND1 (37), forming a positive feedback loop. We reported it here that SND1 promotes the invasive and self-renewal capacity of breast cancer cells and increased sensitivity to Dox, which might be afforded by increased expression of SND1 downstream targets, SMAD2/3/4. Since SMADs are well-known downstream genes of the TGF beta 1 signaling, activation of the SMADs could promote cell invasion, as well as stem-cell like capacity (50). Apparently Linc00668 may bind to SND1 to activate SMAD2/3/4 transcription to cross-talk with TGF beta signaling.

It has also been reported that Linc00668, associating with the PRC2 complex, epigenetically silenced the cyclin-dependent protein kinase inhibitors (CKIs) and promoted cell proliferation in gastric cancer (16). Linc00668 also functioned as a ceRNA to promote tumor progression in several types of cancer (17, 51–54). In this study we observed that Linc00668 promoted breast cancer cell invasion, stem-cell like capacity, and Dox resistance via its binding to SND1.

The exact cause of elevated expression of Linc00668 in breast cancer remained to be identified. E2F1 and STAT3 have been reported to promote the transcription of Linc00668, respectively, in gastric cancer (51) and in lung cancer (16). There is also a large number of predicted binding sites of various transcription factors in the promoter region of Linc00668, including SMADs. Thus, it would be interesting to investigate further whether the activation of SMAD2/3/4 in turn affects the transcription of Linc00668.

In conclusion, we identified the elevated expression of Linc00668 in breast cancer and which was associated with cancer metastasis. Linc00668 promoted breast cancer cell invasion, self-renewal capacity, and resistance to Dox by its association with SND1, further facilitating the expression of downstream genes which considered to be the targets of SND1. Therefore, Linc00668 may serve as a potential therapeutic modulator in breast cancer treatment regimes.

## Data Availability Statement

All datasets generated for this study are included in the article/Supplementary Material.

## Author Contributions

WQ designed this study and analyzed the data. WQ, YZ, and QG performed the experiments. WQ, MW, ZW, PL, and TZ wrote the manuscript. All authors have read and approved the final manuscript and agree to be accountable for the content of the work.

### Conflict of Interest

The authors declare that the research was conducted in the absence of any commercial or financial relationships that could be construed as a potential conflict of interest.
