# Supplementary material for: Linc00668 Promotes Invasion and Stem Cell-Like Properties of Breast Cancer Cells by Interaction With SND1
Source: Front Oncol. 2020 Feb 14;10:88. doi: 10.3389/fonc.2020.00088 (PMC7033544; doi:10.3389/fonc.2020.00088)
Supplement: Supplementary file 1 [file Data_Sheet_1.docx]

Supplementary Material

| Parameter | N | LINC00668 high  expression n (%) | P value |
| --- | --- | --- | --- |
| Lymph node | | | |
| non-metastasis | 26 | 8(30.8) | 0.014(*) |
| metastasis | 28 | 19(67.9) |  |
| Tumor grade | | | |
| 0,1 | 16 | 6(37.5) | 0.016(*) |
| 2,3 | 28 | 22(78.6) |  |
| Age | | | |
| ＜50 | 32 | 15(46.9) | 0.545 |
| ≥50 | 22 | 13(59.1) |  |
| Tumor size（mm） | | | |
| ＜25 | 23 | 12(52.2) | 0.815 |
| ≥25 | 31 | 16(51.6) |  |
| ER status | | | |
| Negative | 22 | 10(45.5) | 0.615 |
| Positive | 32 | 18(56.3) |  |
| PR status | | | |
| Negative | 22 | 11(50.0) | 0.959 |
| Positive | 32 | 17(53.1) |  |
| HER2 status | | | |
| 0,1 | 20 | 10(50.0) | 0.942 |
| 2,3 | 34 | 18(52.9) |  |

**Supplementary Table 1.** Correlation of Linc00668 expression with clinic pathological features in breast cancer patients. *P<0.05 (chi-square test).

**
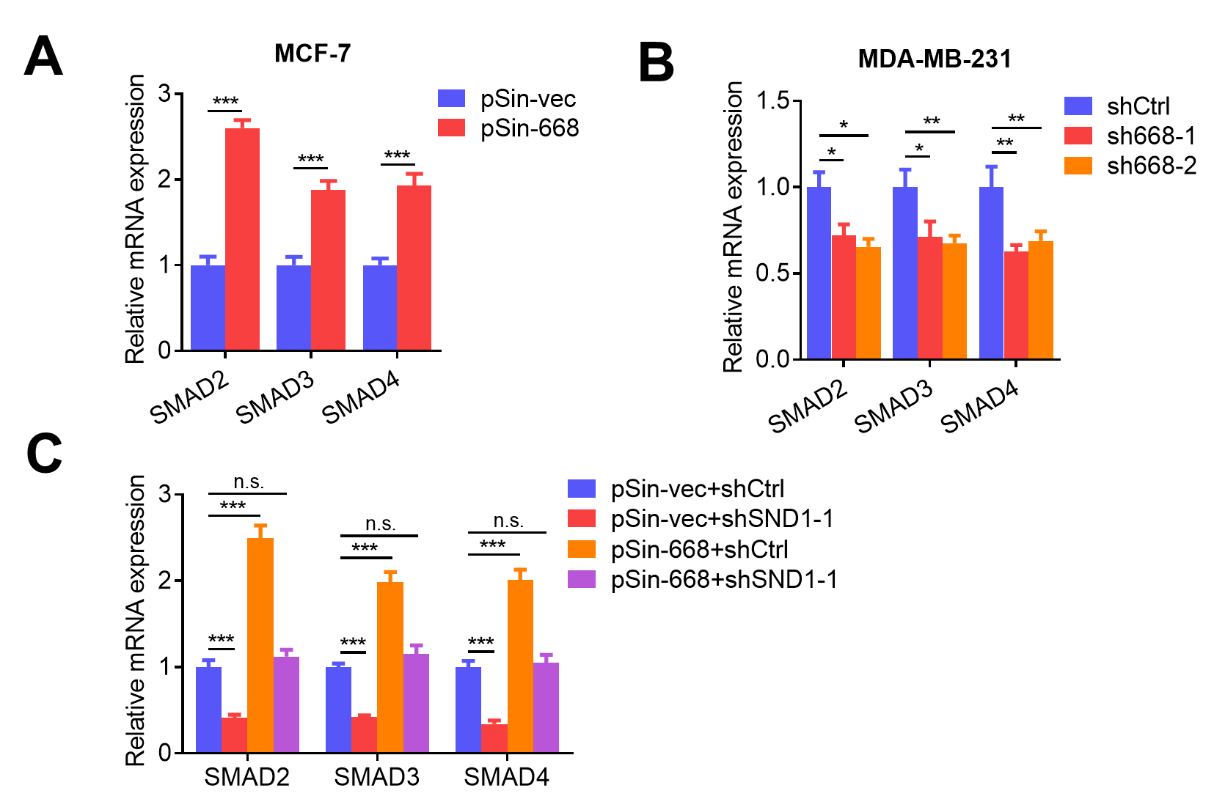
**

**Supplementary Figure 1.** (A-B) Expression of SMAD2/3/4 determined by qRT-PCR in MCF-7 (A) and MDA-MB-231 (B) cells stably transfected with either pSin-668 or shRNA or their respective control vector. (C) Expression of SMAD2/3/4 determined by qRT-PCR in MCF-7 cells stransfected the indicated vectors. *P<0.05; **P<0.01; ***P<0.001; n.s., not significant (Student’s t-test).

**
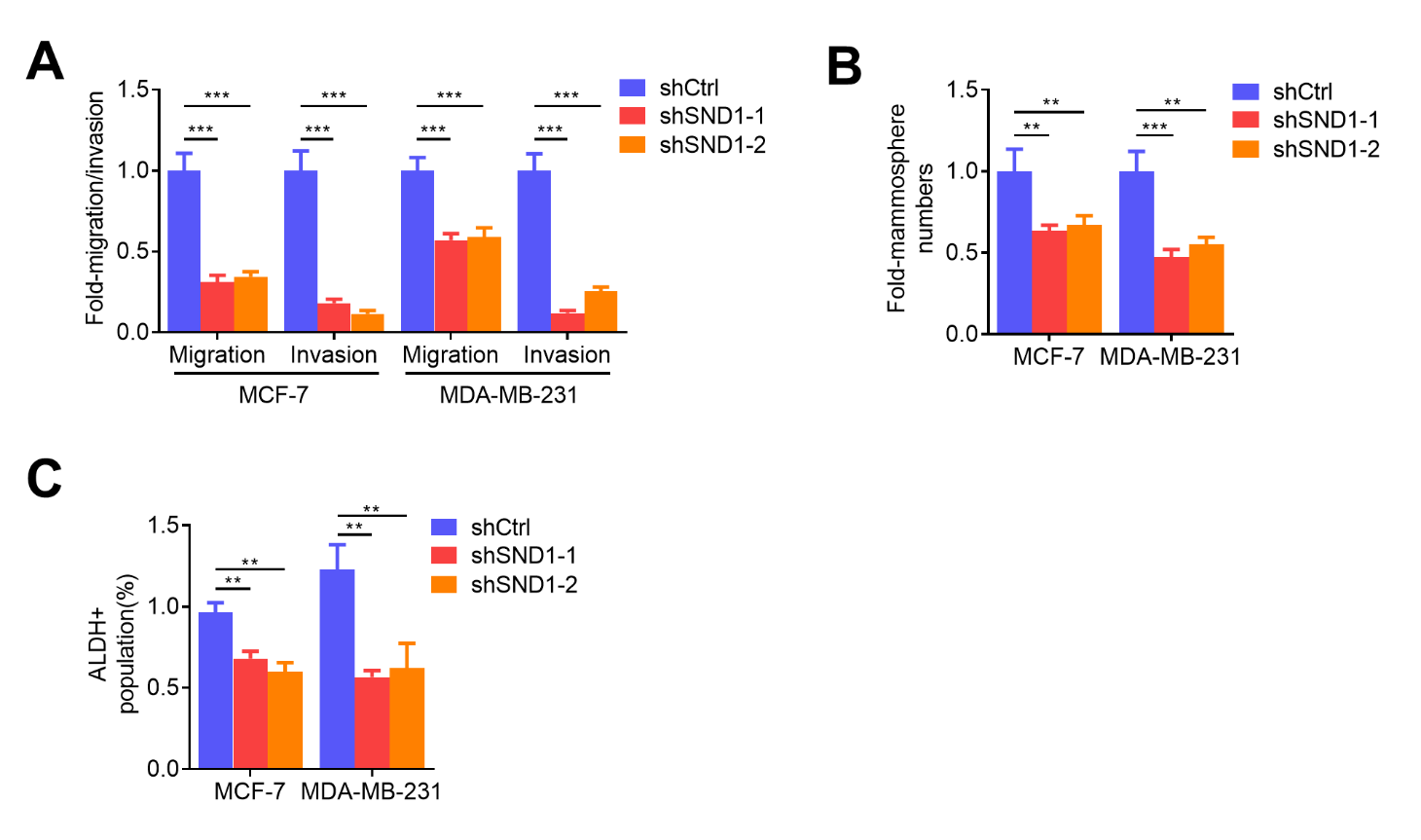
**

**Supplementary Figure 2.** (A) Transwell migration and invasion assays of the MCF-7 and MDA-MB-231 derived cell lines. Quantification of migrated and invaded cell numbers per field is shown as fold changes relative to control cells. (B) Quantification of the mammosphere numbers per field is shown as fold changes relative to control cells. (C) ALDE-FLUOR assay in respective MDA-MB-231 derived cell lines. Results are shown as mean±SD. **P < 0.01; ***P < 0.001 (Student’s t-test).
